# Mass transfer and flow characterization of novel algae-based nutrient removal system

**DOI:** 10.1186/s13068-021-01951-9

**Published:** 2021-04-26

**Authors:** Andreas Heyland, Jordan Roszell, Jeremy Chau, Kevin Chai, Andrew Eaton, Kathleen Nolan, Kyle Madden, Wael H. Ahmed

**Affiliations:** University of Guelph, 50 Stone Rd E, Guelph, ON N1G 2W1 Canada

## Abstract

**Background:**

Recirculating aquaculture systems (RAS) are an essential component of sustainable inland seafood production. Still, nutrient removal from these systems can result in substantial environmental problems, or present a major cost factor with few added value options. In this study, an innovative and energy-efficient algae based nutrient removal system (NRS) was developed that has the potential to generate revenue through algal commercialization. We optimized mass transfer in our NRS design using novel aeration and mixing technology, using air lift pumps and developed an original membrane cartridge for the continuous operation of nutrient removal and algae production. Specifically, we designed, manufactured and tested a 60-L NRS prototype. Based on specific airlift mixing conditions as well as concentration gradients, we assessed NRS nutrient removal capacity. We then examined the effects of different internal bioreactor geometries and radial orientations on the mixing efficiency.

**Results:**

Using the start-up dynamic method, the overall mass transfer coefficient was found to be in the range of 0.00164–0.0074 ${\mathrm{s}}^{-1}$, depending on flow parameters and we confirmed a scaling relationship of mass transfer across concentration gradients. We found the optimal Reynolds number to be 500 for optimal mass transfer, as higher Reynolds numbers resulted in a relatively reduced increase of mass transfer. This relationship between mass transfer and Reynolds number is critical to assess scalability of our system. Our results demonstrate an even distribution of dissolved oxygen levels across the reactor core, demonstrating adequate mixing by the airlift pump, a critical consideration for optimal algal growth. Distribution of dissolved gases in the reactor was further assessed using flow visualization in order to relate the bubble distribution to the mass transfer capabilities of the reactor. We run a successful proof of principle trial using the green alga *Dunaliella tertiolecta* to assess mass transfer of nutrients across the membrane and biomass production.

**Conclusions:**

Manipulation of the concentration gradient across the membrane demonstrates a more prominent role of airlift mixing at higher concentration gradients. Specifically, the mass transfer rate increased threefold when the concentration gradient was increased 2.5-fold. We found that we can grow algae in the reactor chamber at rates comparable to those of other production systems and that the membrane scaffolds effectively remove nutrients form the wastewater. Our findings provide support for scalability of the design and support the use of this novel NRS for nutrient removal in aquaculture and potentially other applications.

## Background

With the growing demand for sustainable food production, land-based aquaculture is becoming an increasingly relevant alternative to existing approaches [1, 4]. In recent years, inland aquaculture has grown rapidly as it presents a more environmentally sustainable way to produce seafood compared to aquaculture using natural water bodies [14]. In situ pond and pen growth methods are known to experience frequent fluctuation in the surrounding water quality which can disturb the ecological balance required to maintain healthy growth conditions for aquatic livestock [2, 3]. In comparison to these conventional aquaculture systems, inland recirculating growth systems can be operated under controlled conditions at high stock densities. Furthermore, inland aquaculture systems reduce area footprint as well as the overall water consumption of the aquaculture facility per unit lifestock or biomass produced [46]. Nevertheless, inland aquaculture requires high levels of maintenance and conventional methods for nutrient removal (nitrogenous waste—NO_2_, NO_3_, NH_4_, phosphate—PO_4_ and CO_2_) add to the production costs without adding revenue. Nutrient accumulation in RAS is attributed to decomposition of excess feed supplied to the system and also from the excretory and digestive by-products released by the aquatic livestock into the system. Removal of these compounds is crucial, as high concentrations of nitrogenous and phosphate compounds can impact the growth and health of the aquatic livestock being reared and CO_2_ increase in RAS can lead to acidification [46].

There are several methods currently implemented in recirculating aquaculture systems to remove nutrients from the water. Typically, this includes the removal of ammonium and other waste products (NO_2_, NO_3_, NH_3_ and PO_4_). In order to reduce water loss, the sludge is typically thickened during this process which limits its downstream applications. These systems are also operated with smaller treatment volumes when compared to outdoor recirculating aquaculture systems [48]. For outdoor systems, high rate algal ponds and wetlands have been employed [48]. With these systems, the production of seafood can be combined to grow valuable biomass. However, these approaches and designs are highly dependent on the surrounding climatic conditions and require large treatment areas [48].

Microalgae are highly effective at removing nutrients from water and present an important alternative to bacterial denitrification systems [12]. As photosynthetically active organisms, they can not only remove nitrogenous and phosphorous products but also CO_2_, which in turn is required for photosynthetic activity. Moreover, microalgae grow and clonally replicate within short time periods and are an extremely diverse group of organisms that provide a broad selection of species that can be acclimated to almost any effluent [10, 11, 36]. More importantly, microalgal biomass has potential commercial applications, such as their use in the cosmetics, pharmaceutical and agricultural industries [22, 38, 40]. For example, several species of microalgae have been implemented in a variety of cosmetic products, providing companies with a relatively cheap source of valuable molecules to be used in hair and skin care products [40, 43]. Microalgae species are also utilized to produce nutrient supplements; Specifically, *Spirulina*, *Chlorella,* and *Dunaliella,* have been shown to contain relatively high concentrations of carotenoid and vitamin B compounds, which are important for the health and well-being of several animals [22, 44]. Furthermore, much research has explored microalgae as a dietary supplement for humans. *Spirulina, Chlorella, Dunaliella, Haematococcus, Schizochytrium,* and *Isochrysis* have been shown to be nutrient rich and can be used as microalgae-based supplements for human consumption [9].

Within the agricultural and environmental sector, species such as *Chlorella vulgaris* and *Spirulina platensis* have been used as bio-fertilizers [10, 11] and some species have proven useful for phycoremediation of heavy metals, such as zinc (Zn^2+^) and copper (Cu^2+^), in water and soil [19]. Recent studies of Zhu [50] and Lee [30] have shown promising results for utilizing microalgae to sequester CO_2_ into algal biomass, convert nitrogen and phosphate to biomass via cellular incorporation and to enrich the amount of dissolved oxygen in the water via the metabolic conversion of NADP^+^ to NADPH [7]. In aquacultural facilities, microalgae have been implemented as a nutritionally complete food item for aquatic larva and livestock [18].

**Fig. 1:**
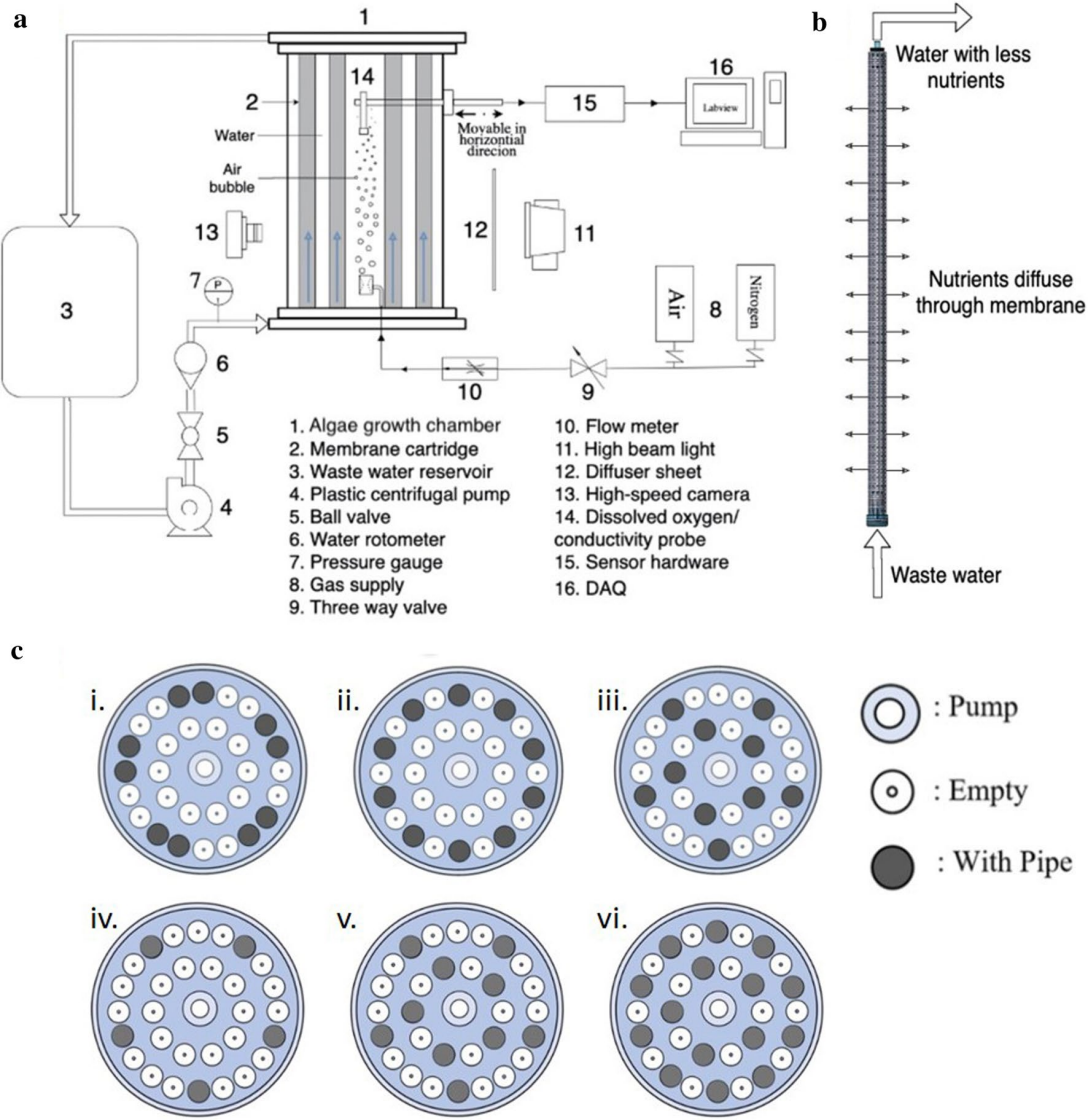
Overview of photobioreactor design and experimental setup used in this study. **a** System schematic with algal growth chamber (1), wastewater reservoir (3) and Data Acquisition Options (DAQ) (16). **b** Close-up of membrane cartridge unit for nutrient removal. Note that the reactor can accommodate up to 30 of these cartridges inside the chamber. **c** Experimental setup of nutrient removal cartridge arrangements in this study. (i.–iii.) Experiments 1–3; (iv.–vi.) Experiments 4–5

One challenge of using microalgae in nutrient removal is their effective separation from the water that is used to culture organisms. Therefore, we developed a microalgal photobioreactor prototype (Fig. 1) that uses dialysis membrane to separate the RAS water from the algal culturing system in order to remove excess CO_2_ and nutrients without algal permeation. We also tested the effectiveness of a new airlift technology for mixing and enhancing the reactor’s capacity for nutrient removal. We analyzed these innovations rigorously using non-dimensional parameters to scale the prototype reactor to an industrial scale. Finally we provide a proof of principle for our prototype and show that green algae can be grown in our system at rates comparable to commercial production systems.

## Results

### Aeration and mixing

**Fig. 2:**
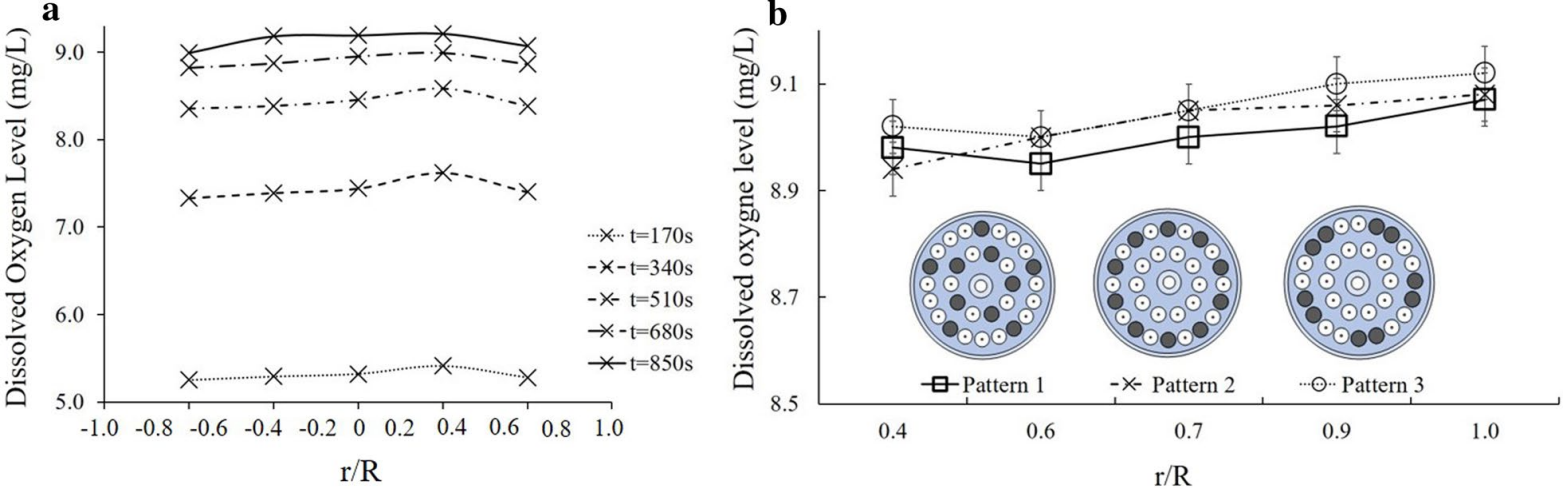
Radial profiles of dissolved oxygen levels within the reactor chamber. **a** Radial profile across the full diameter of the reactor chamber without membrane cartridges measured at five time points from 170 s up to saturation at 850 s. Note that numbers are in reference to reactor chamber midline. **b** Radial profile across one half of the reactor chamber with three different membrane cartridge patterns present (used cartridges in dark blue)

Experiments 1 and 2 were conducted in order to test the mixing and aeration in the reactor chamber as well as oxygen mass transfer under different operating conditions. In Experiment 1, we created horizontal profiles at five time points and detected only minimal variation in DO levels across the diameter of the reactor chamber (Fig. 2a). The center had a slightly higher dissolved oxygen level because the spiral airlift pump is located in that position. Empirically measured O_2_ concentrations at equilibrium matched those calculated using Henry’s law (Fig. 2a). When testing DO level profiles for different membrane cartridge configurations, we found comparable patterns between the configurations as well as to the empty chamber (Fig. 2b).

**Fig. 3:**
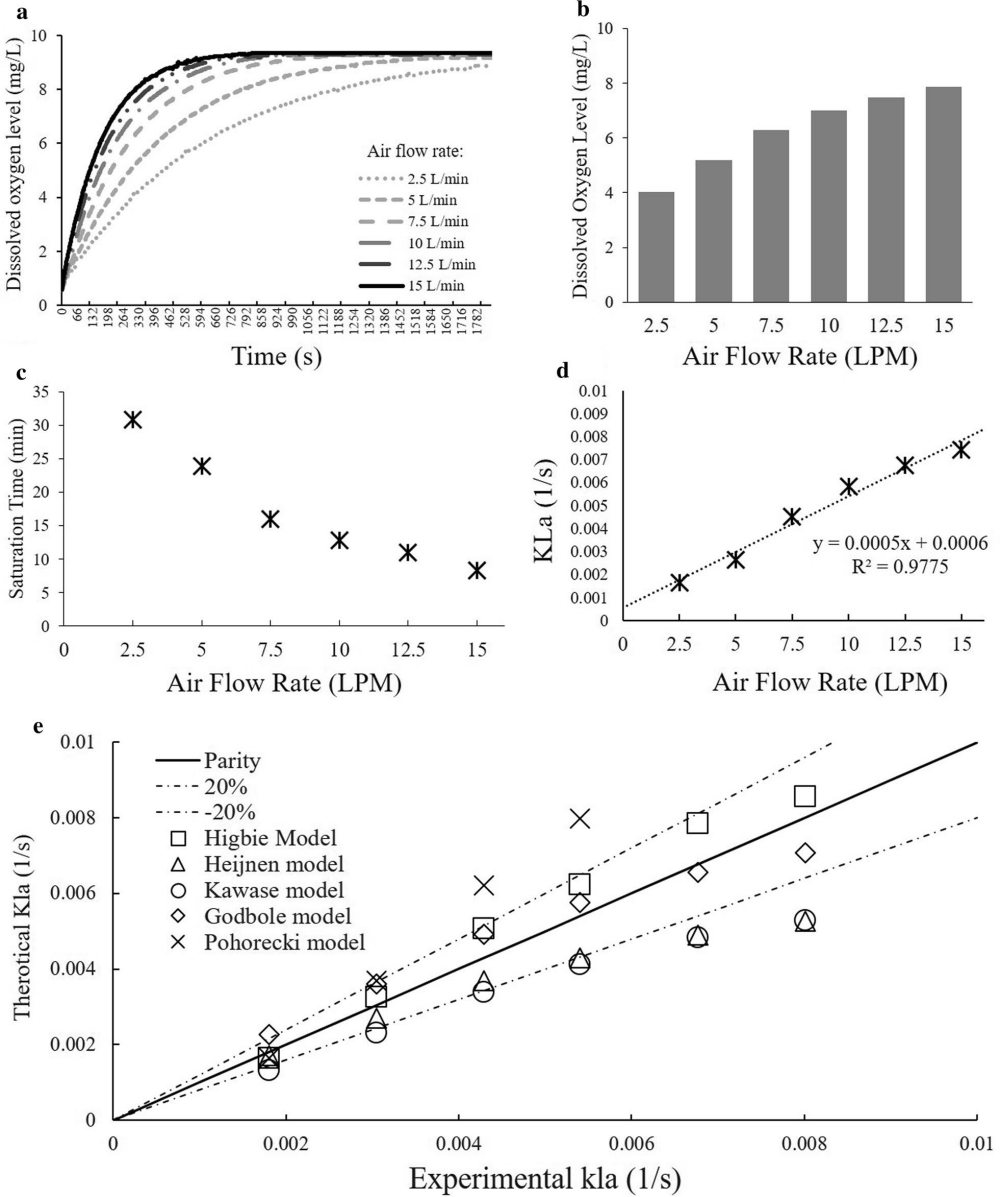
Dissolved oxygen (DO) level at various gas flow rates within the reactor chamber. **a** DO as a function of time and six air flow rates. **b** Average DO as a function of air flow rates. **c** Saturation time (min) as a function of air flow rates. **d** Calculated oxygen mass transfer rate as a function of air flow rate with linear regression model. **e** Fitting of existing mass transfer models to the existing data

In Experiment 2, we tested the consequences of different superficial gas flow rates ranging from 2.5 to 15 L/min in the bioreactor in DO levels and oxygen mass transfer (Fig. 3). Overall, higher gas flow rate reach saturation faster (Fig. 3a–c). For instance, the dissolved oxygen level reached equilibrium at around 500 s (8.3 min) when the air flow rate was at 15 L/min, whereas it required around 957 s (15.9 min) to reach equilibrium when the air flow rate was at 7.5 L/min (Fig. 3a). The gas flow rate has a strong influence on the mass transfer ability of an air–water system (Fig. 3d) and the overall mass transfer coefficient also has a positive linear correlation with an *R*^2^ value of 0.98 across the air flow rates tested. For example, when the air flow rate is at 2.5 L/min, the overall mass transfer is 0.001638 s^−1^, and as the air flow rate rises to 15 L/min, the overall mass transfer coefficient increases to 0.007413 s^−1^.

We used the mass transfer coefficient data to compare our reactor column to several other mass transfer systems within bubble columns (Table 2). With all the mass transfer models tested, only the top five best fit correlations were selected for comparison and are shown in Fig. 3e. All of these correlations are within the 40% error window and most of them lie within a 20% error range, with the exception of two datapoints for the Pohorecki model. To further study the models, we implemented the mean absolute percentage deviation (MAPD—Eq. 1) in order to assess the accuracy of the correlation between the model and our experimental results. Both the Higbie model and the Godbole model resulted in an MAPD value that is less than 15%, which indicates that the model is a fairly good fit to the parameter of this project:

1 $$\mathrm{MAPD}=\frac{100\%}{n} \sum_{t=1}^{n}\left|\frac{{{A}_{t}-F}_{t}}{{A}_{t}}\right|.$$

**Fig. 4:**
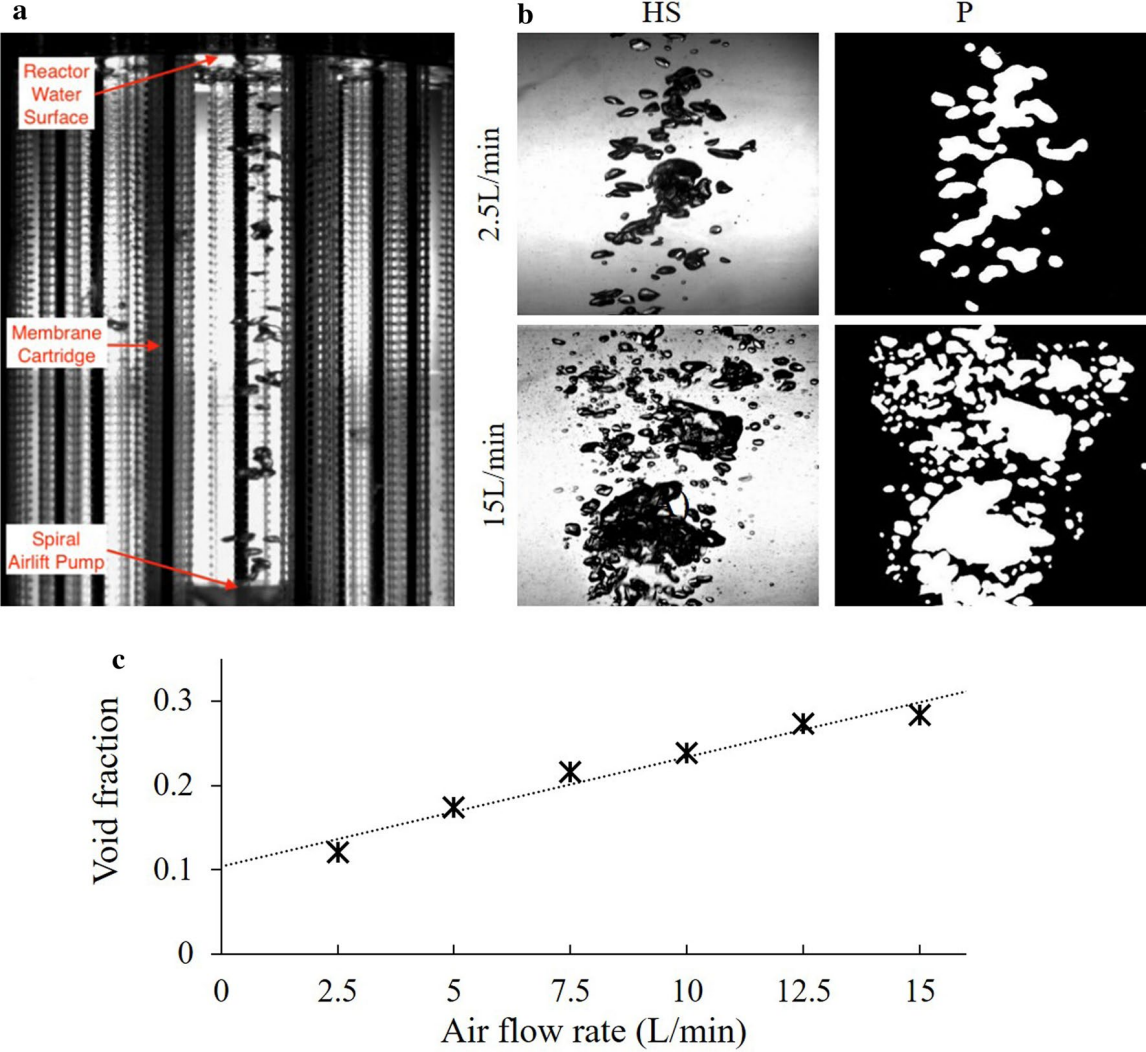
Multiphase flow visualization during reactor operation (**a**) and empirical assessment of air void fraction with the reactor chamber (**b**, **c**). **a** Image of reactor chamber using high-speed camera setup. **b** Representative high-speed frames of lowest (2.5 L/min) and highest (15 L/min) air flow rates with processed images used for analysis. Complete set of representative images across the entire range can be found

In Experiment 3, we characterized the bubble pattern and void fraction in response to the specified gas flow range. Figure 4 shows that the flow pattern within the reactor remained in bubbly flow and low gas flow. However, as the gas flow rate increased, the bubbles started to coalesce and form bigger bubbles that spread out further (Fig. 4). Furthermore, the void fraction of gas in the reactor increases with increasing gas flow as expected, as there is a larger portion of gas in the system. At a gas flow rate of 2.5 L/min, the void fraction is around 12%, and as the flow rate rises to 15 L/min, the void fraction reaches around 28%. These results indicate that the relationship is not linear and that the saturation of the void fraction occurs at higher gas flow rates.

### Membrane cartridge mass transfer characteristics

**Fig. 5:**
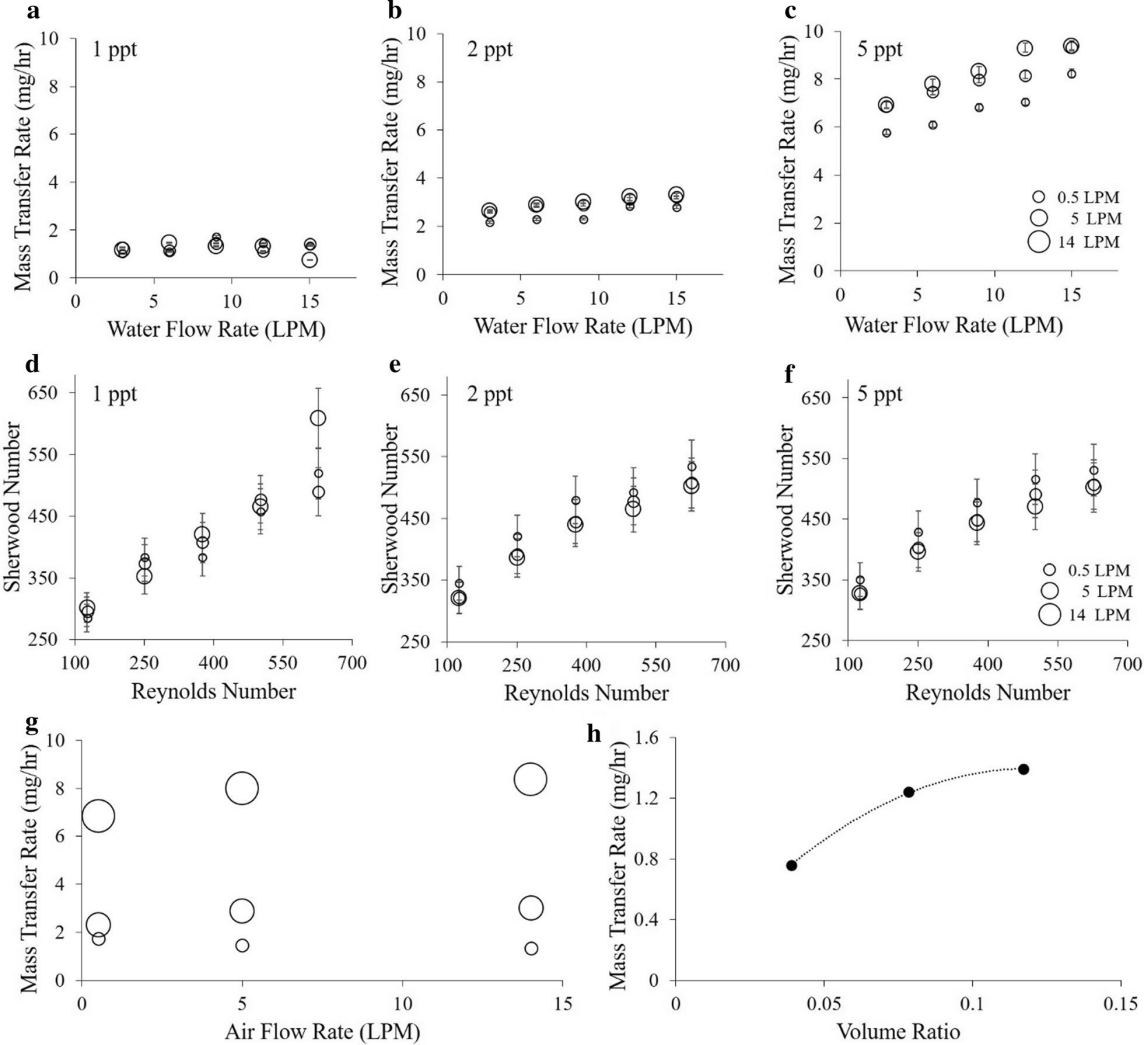
Salt mass transfer rates as a result of five water flow rates and three air flow rates across three salinity gradients (**a**–**c**). Calculated non-dimensional plots based on mass transfer values across three salinity gradients (**d**–**f**). **g** Summary of mass transfer rates as a function of three air flow rates (0.5 LPM, 5 LPM and 14 LPM) and three salinity gradients (1 ppt, 2 ppt and 5 ppt—represented by increasing sized circles) with membrane cartridges present. **h** Mass transfer rate across membrane cartridges as a function of volume ratio of cartridges occupied

Comprehensive experiments were conducted to characterize the effects of various operational parameters on the mass transfer rates for the prototype algae bioreactor. Figure 5a–c, g illustrates the increase of mass transfer as a function of the salt gradient. Quantitatively, the mass transfer scales directly with the concentration gradient. When decreasing the concentration gradient from 5 to 2 ppt (reduction factor of 2.5) the average mass transfer rate reduces from 7.72 to 2.81 mg/h, corresponding to a reduction factor of 2.7. Similarly, reducing the initial salt gradient from 2 to 1 ppt, the average mass transfer rate dropped to 1.26 mg/h, for a reduction factor of 2.21.

In addition, these experiments show that both water flow rate through the membrane cartridge as well as air flow rate in the reactor column affect the mass transfer rate. However, the impact of air flow is minimal at low salinity gradients and becomes more substantial at higher gradients. Specifically, we did not measure any substantial effect of water and air flow on mass transfer rate when the salinity gradient was 1 ppt. At 2 ppt, mass transfer rate increased slightly with both water and air flow. The strongest impact of water and air flow was observed at 5 ppt with the highest mass transfer rates resulting from high water flow rate (15 LPM) and air flow rate (14 LPM). When we tested the impact of increased membrane surface area on mass transfer, we identified a surface area-to-volume ratio of 0.18 to produce maximum mass transfer rates (Fig. 5h).

When we transformed these mass transfer rates into dimensionless parameters (Sherwood number and Reynolds number—Fig. 5d–f), we found that mass transfer rates scale proportionally across salinity gradients. Additionally, increasing the air flow rate at a given Reynolds number and concentration reduced the Sherwood number. However, the Sherwood number has a tendency to plateau at higher Reynolds numbers (~ Re = 500).

### Growth characteristics for *D. tertiolecta* in the photobioreactor

**Fig. 6:**
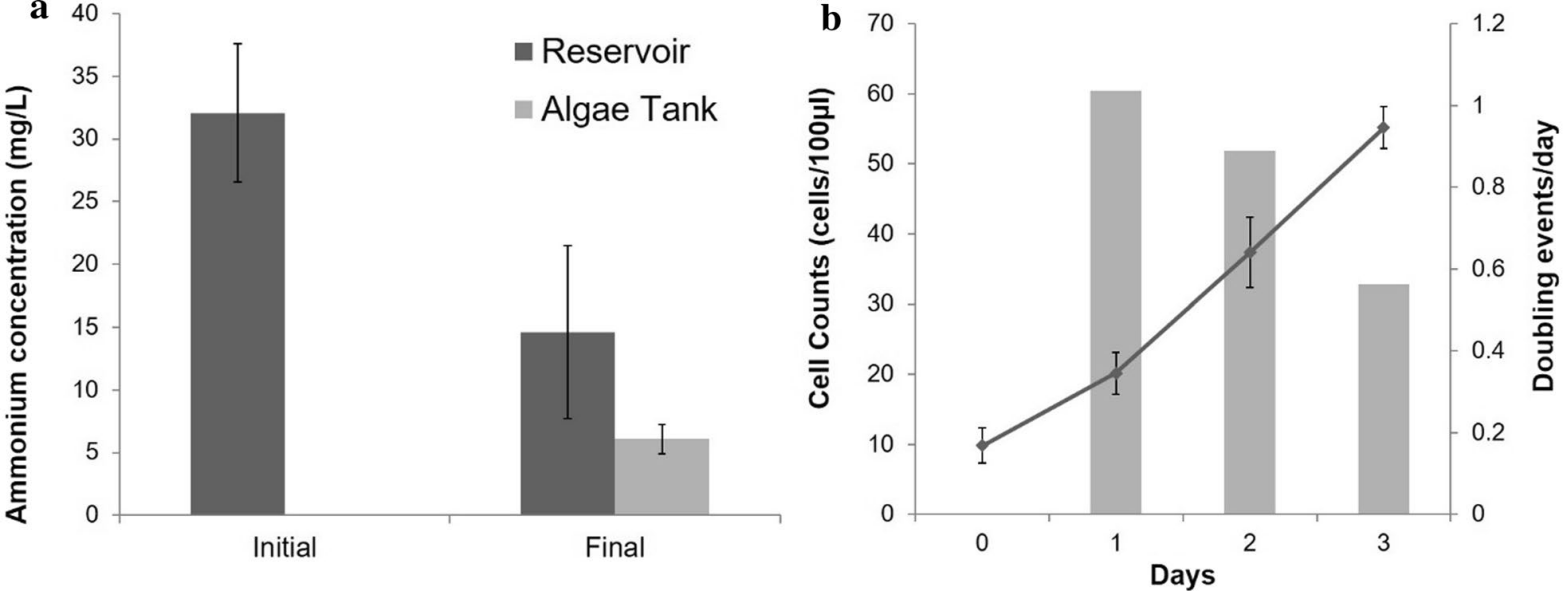
Production of the green alga *Dunaliella tertiolecta* in our novel photobioreactor prototype. **a** We used ammonium as a representative and measureable nutrient in our experiments to compare mass transfer characteristics across the membrane scaffolds over a 5-h period, resulting in a mass transfer rate of 1.2 mg/h. **b** We then added nutrient medium to the reservoir and grew *D. tertiolecta* in the algal tank using standard parameters (see text for details). Based on cell density increase (cell counts—line graph) as well as estimated doubling rates (bar graph), we found that *D. tertiolecta* growth in our system is comparable to growth reported in the literature. These experiments provide preliminary evidence for the nutrient removal and biomass production capabilities of our system. Error bars indicate 2 SE of the mean

Ammonium mass transfer trials at 5 LPM air flow rate and ammonium concentration representing a standard nutrient gradient (Fig. 6a) resulted in a mass transfer of 1.2 mg/h and a mass transfer coefficient of 0.06 (h^−1^). This mass transfer is comparable to our salt transfer rates shown in Fig. 5 and suggests that nutrients can pass through our membrane scaffolds at comparable rates as assessed by salt gradients. In order to estimate algal dry biomass production in our system, we compared cell counts over a 3-day period and doubling events in our reactor to data from the literature [45]. We found that under standard conditions (4 LPM) the estimated dry biomass production in our study would result in 0.036 g/L/day.

## Discussion

We conducted a series of experiments in order to assess basic mixing and aeration effects on mass transfer rates within a novel photobioreactor. We designed this photobioreactor for nutrient removal in recirculating aquaculture systems. For simplicity, we conducted experiments using salt gradients initially as they can be measured accurately in real time. This allowed us to assess the scalability of our nutrient removal system. We also provide experimental data on nutrient transfer and biomass production as a proof of principle and to show that this nutrient removal system can be used in a recirculating aquaculture system.

The photobioreactor design we developed consists of three interconnected spaces with diffusion of ions and gases (Fig. 1): (1) diffusion of ion and water across the membrane cartridge; (2) diffusion of gases within the reactor chamber and (3) diffusion of gases from air bubbles created by the airlift pump into the reactor chamber. As the photobioreactor will be operated with algae in the reactor chamber and waste water flowing through the membrane cartridge, the relevant mass transfers are: (1) oxygen transfer from reactor chamber into waste water stream; (2) CO_2_ transfer from waste water stream into the reactor chamber, (3) CO_2_ transfer from bubble column into the reactor chamber, and (4) nutrient transfer from waste water stream into the reactor chamber. The measurements presented here provide critical insight into all of these potential mass transfer rates and therefore the efficient operation of this reactor design.

### Mixing and mass transfer properties across reactor compartments

Proper mixing of water, algae and gases in the reactor chamber is critical to accomplish maximum growth rates [47]. We were able to confirm a homogenous distribution of oxygen within the reactor under the operating conditions tested, including the addition of membrane cartridges that can potentially obstruct mixing. Furthermore, the oxygen saturation curves we generated across a range of air flow rates show that even under the lowest air flow rate we can reach saturation after 30 min. This is a sufficient time frame for operating this algal reactor.

With respect to the oxygen mass transfer models we tested, both the Higbie [25] model and the Godbole [21] model resulted in a fairly good fit. The Higbie [25] model, also known as the penetration theory, has been widely used for finding the mass transfer in a bubble column. The Godbole [21] model is an empirical equation that estimates the ${k}_{L}a$ value in the bubble column by establishing the relationship between the ${k}_{L}a$, the superficial gas velocity, and the fluid viscosity. The first part is the mass transfer coefficient ${k}_{L}$ which is related to the mass transfer rate in the diffusion process, and the second part is the effective interfacial area $a$ which is related to the area of the interface where the reaction takes place. The constant and the exponent can be changed according to the flow conditions and the fluid used in the system. This equation can also be used if a non-Newtonian fluid is used. When increasing the volume of the growth chamber of the reactor prototype, this model can also be used to predict the required air flow rate to accomplish appropriate mixing.

Characterizing the multiphase flow mixture inside the bioreactor body with the membranes installed is an important element to the design of the overall system. Key properties that are needed to characterize the mixing include bubble shape and size, rising velocity, turbulence and induced secondary flows [16]. A flow visualization was used to relate the bubble distribution and the void fraction in the bioreactor system. As the air flow rate increases, the size of the bubbles and the amount of bubbles also increase. Therefore, the void fractions are strongly correlated with bubble size and the flow pattern in the reactor. We observed coalescence of bubbles at higher air flow rates, explaining the slight saturation of the void fraction. Typically, perforated plates are used in photobioreactors to break up coalesced bubbles in taller reactors [42]. Still, in our experiments, these larger bubbles can also rise faster than smaller bubbles, contributing to higher turbulence and mixing within the chamber. We also observed that in general, the dissolved oxygen concentration across the bioreactor diameter was evenly distributed. For each time interval, the differences in the dissolved oxygen concentration were within 0.2 mg/L. This is mainly because the spiral airlift pump creates a vortex in the bioreactor, so the liquid inside the bioreactor is well mixed, and thus the dissolved oxygen level is evenly distributed. We also noticed a higher level of spiral mixing at these high flow rates which will be a critical component to consider when the reactor chamber is expanded in volume, while maintaining a similar diameter. In this case, larger bubbles and spiral injection will ensure proper mixing along the entire length of the reactor. This is a distinct advantage of our design over existing spargers that typically have reduced back mixing at low gas flow rates [26].

Our experiments were designed to assess the volumetric mass transfer behavior of a bioreactor using a spiral airlift pump. Our multi-dimensional assessment of air/water flow rate and salinity gradient revealed an increase in mass transfer with increased flow rate inside the membrane tube. These results are consistent with theory in that the flow boundary layer inside the tube is reduced at higher velocities, leading to relatively higher salt ion concentrations in proximity of the membrane and therefore increasing the diffusion rate. These ions are then transported through the membrane via diffusion at low pressures (< 34.5 kPa) and a combination of diffusion and convection at higher pressures. This effect is enhanced by air injection, as it disrupts boundary layer effects on the reactor chamber side of the membrane. Still, operating the reactor at high (5 LPM and more) air injection rates resulted in irregularities in mass transfer, suggesting that air flow rates should be kept minimal to optimize mass transfer at Reynolds numbers around 500 (the Re number we estimate to be optimal for operating our reactor). Varying the flow rate of the recirculating water affects the velocity in proximity to the wall of the membrane scaffolds. In our case, the desired (efficient and scalable) conditions are characterized by a boundary layer mechanism for optimum mass transfer at the inner wall. These conditions were verified by running a combination of factors that typically influence the mass transfer (flow rate, gradient, etc.). We found that higher Reynolds numbers are not resulting in more beneficial mass transfer coefficients. Therefore, we conclude that it is more efficient, from an energy perspective, not to drive higher velocity flow, which in turn would require more operating power. Also, the mass transfer at the outer wall of the member is characterized by the turbulence generated by the two-phase bubbly flow that creates the mixing effect. This was investigated by injecting different air flow rates and the optimum values were obtained. Knowing the required power for air injection is minimum and therefore having optimum Reynolds inside and enhancing transfer at the outer wall is quite reasonable for a reactor design in order to be scaled for commercial purposes. We hypothesize that turbulent flow at higher levels of air injection causes pressure fluctuations at the fresh water–membrane interface and disrupts the diffusion of salt ions through the membrane. It has also been shown in previous studies that turbulent flow can generally enhance growth rates of algae in photobioreactors [5, 27].

### Reactor scaling and aquaculture implementation

In recirculating aquaculture operations, the daily nitrogen budget of a production system is typically known and allows to make specific predictions about nutrient removal requirements based on acceptable concentration of nitrate and other nutrients in the system [34, 41]. Our trials with salt water gradients revealed a predicted mass transfer rate from the membrane cartridge into the reactor chamber of 0.5 kg/24 h/reactor, assuming optimal operating conditions as they were determined in this study. While this value is based on the small 60-L prototype reactor and a 50% membrane surface capacity, it allows to make certain predictions about the scalability and application of our new reactor technology. Specifically, the use of dimensionless parameters, Rn for fluid dynamics and Sherwood number for mass transfer, allows us to extrapolate the reactor properties beyond the dimensions of the current prototype. This is a crucial aspect of our study, as scalability can frequently be a difficult aspect of photobioreactor design [47].

Aeration of the reactor chamber is crucial for mixing; however, other parameters such as bubble size and velocity can impact the growth and health of algae in the reactor. For example, previous work has shown that lipid production by *Chlorella* sp. is negatively impacted by larger bubble sizes in the reactor, likely due to physical stress on the cells [49]. Larger bubbles also result in poorer mass transfer of oxygen and CO_2_ in photobioreactors [47], a problem that can be addressed by breaking up larger bubbles within the reactor column using perforated plates [42]. Furthermore, the mixing also allows more efficient light usage inside the reactor chamber and therefore algal growth at higher concentrations [47]. This is a critical consideration as space can be limiting in algal production settings. Our trial experiments with *D. tertiolecta* resulted in growth characteristics comparable to other reactor systems [45]. Future experiments will be conducted to optimize growth based on specific bubble pattern.

Vertical-column photobioreactors are generally considered easy to operate and can be used for large-scale culturing [39]. With the addition of bubble column and airlift technology, as in our design, these reactors can achieve similar algae production rates as narrow tubular reactors by using less space and resulting in less clumping and settlement inside the tubes [15]. Our preliminary trial with *D. tertiolecta* resulted in minimal fouling of the tubes and we did not observe excessive settlement of algae inside the reactor column. While these results are promising, future experiments will have to assess the long-term performance of the reactor with respect to fouling. Additionally, our reactor design has the ability to transfer nutrients efficiently from a waste water stream into the algae compartment, without getting the algae directly into contact with the waste water. This latter design component is very suitable for inland aquaculture as it significantly reduces the risk of cross contaminating production systems. Our estimated dry biomass production of 0.036 g/L/day is comparable to other systems. However, the biomass production can be potentially significantly increased by optimization of nutrient conditions, injection pressure and CO_2_ addition. These parameters will be assessed in future studies. We also found that nutrient transfer across the membrane is not limiting algal growth in our experiments. However, under aquaculture conditions, this will depend on nutrient production in the recirculating water system.

## Conclusion

In conclusion, we designed, built and tested a new NRS with state of the art bubble column and airlift technology for the continuous operation of nutrient removal and algae production. The NRS nutrient removal capacity was assessed based on radial concentration gradients. Also, the concentration of dissolved gases in the reactor was assessed using flow visualization in order to relate the bubble distribution to the mass transfer capabilities of the reactor. The optimal Reynolds number was found to be 500 for optimal mass transfer, and this was found to be a critical parameter to assess scalability of our system. The results also demonstrate an even distribution of dissolved oxygen levels across the reactor core, demonstrating adequate mixing by the airlift pump, a critical consideration for optimal algal growth. Moreover, the experimental data for oxygen and salt mass transfer suggest that the present design is highly applicable to inland aquaculture settings, where excess nutrients need to be removed from the production system in order to ensure the healthy growth of animals. Specifically, the mass transfer rate increased threefold when the concentration gradient was increased 2.5-fold. Our growth test with the green alga D. tertiolecta provides evidence that the mass transfer parameters developed in this study translate into biomass production comparable to other reactor systems. Importantly, the nutrient supply across the membrane does not limit algal growth in our prototype. These findings provide support for scalability and the use of this novel NRS for nutrient removal in aquaculture.

## Materials and methods

### Experimental setup

The photobioreactor is divided into three main subsystems that, when operating together, can remove molecules of small molecular size, including nutrients, from wastewater supply streams (Fig. 1a, b). These subsystems include: (1) the algae reactor body, (2) the wastewater recirculation loop (Fig. 1a), and (3) the membrane cartridge (Fig. 1b). The prototype algae column consists of a 60-L cylindrical acrylic tank with an inner diameter of 0.30 m and a height of 0.63 m. The design of the membrane cartridge, shown in Fig. 1b, allows nutrients to move to the algae chamber from an external wastewater supply. The membrane cartridge is designed to facilitate the transfer of these compounds and CO_2_ into the algae chamber via diffusion driven by concentration and pressure gradients. Additionally, as the algae metabolize CO_2_ and nutrients to produce O_2_ via photosynthesis, the dissolved oxygen content in the algae chamber will rise and will ultimately be transferred to the flow stream inside the membrane cartridge. Hence, the primary function of the algae reactor body is to provide a suitable environment for the algae to grow. As a substitute for traditional mixing methods, such as mechanical paddles and motors which can require maintenance due to algal caking, the prototype photobioreactor makes use of multiphase flow technology and we implemented a spiral injection airlift pump developed by FloNergia™ (Fig. 1). This system injects air into the algal chamber which creates a three-phase mixture of water, air, and algae. As the bubbles rise, they induce a circulation effect to enable effective mixing of algae. Additionally, the water chemistry can be controlled by changing the composition of the gas that is injected in order to optimize the algae growth rate. For example, the injection of CO_2_ may complement existing pCO_2_ which may be beneficial for some algae species [35].

### Experimental design

#### Aeration and mixing experiments

We conducted three experiments to gain insights into aeration and mixing within the reactor chamber. Specifically, we manipulated air flow rates under a range of internal reactor geometries and measured dissolved oxygen (DO) concentrations inside the chamber. From this data, we were able to calculate oxygen mass transfer rates. Additionally, we conducted two-phase flow experiments using high-speed video acquisitions of the bubble column created in the reactor chamber and analyzed gas void fraction as a function of air flow rate using high-speed video analysis.

For Experiments 1 and 2, we reduced DO in the chamber using nitrogen until we reached levels of approximately 0.8 mg/L. We then sparged oxygen into the system through an airlift pump, designed to provide mixing using the swirling effect of bubble dynamics, until saturation was reached. Based on Henry’s law, we calculated the saturated dissolved oxygen concentration to be roughly 9.1 mg/L at room temperature (21 °C) and standard pressure. This agreed with the experimental data, which found that the dissolved oxygen concentration at equilibrium was around 9.1 mg/L. DO levels were measured continuously with a temporal resolution of one second using a galvanic dissolved oxygen probe with an accuracy of $\pm$ 0.05 mg/L.

For each test, the total water flow rate as well as the air injection flow rate was held constant using a centrifugal pump and air regulator, respectively. The water and air flow rates were measured using a FL50000 rotameter (OMEGA) and OMEGA FMA 1612A flowmeter (OMEGA), respectively. The tank and reservoir conductivity were each measured using an Atlas Scientific K1.0 E.C probe connected to LABVIEW sampled at 1 Hz. The reservoir water pressure was measured using a Winters PFQ pressure gauge. Additionally, the range and corresponding error for each instrument are shown in Table 1. We encountered noise in our measurements due to air bubbles in the reactor that interfered with the dissolved oxygen probe. To reduce the noise from the data, a Savitzky–Golay filter was applied. This filter has a built-in Matlab function and was used to smooth out the signal using a least square regression.

**Table 1:** Instrumentation range and uncertainty

Instrument	Range	Error
OMEGA FL 50000 Rotameter	2–20 LPM	2%—full scale
OMEGA FMA 1612A Flowmeter	0–500 SLM	0.8% rdg + 0.2% FS
Atlas Scientific K 1.0 E.C. probe	5–200,000 μS/cm	2%
Winters PFQ Pressure Gauge	0–30 PSI	2.5%—full scale

##### Experiment 1: DO distribution as a function of reactor geometry

We assessed the distribution of DO in a cross section of the reactor chamber with and without membrane cartridges present. The gas flow rate for all trials was set at 10 L/min. The radial profile of DO measurements provides a representation of the mass transfer phenomenon occurring across the bioreactor’s diameter. The bioreactor’s inner diameter is 30 cm and we assessed five data points across this diameter. The radial profile was recorded every 170 s. Additionally, ten 5-cm acrylic pipes were inserted into the bioreactor in different configurations. The goal was to determine whether the internal geometry of the bioreactor would affect mixing. The various patterns are illustrated in Fig. 1ci–iii. These patterns were chosen because they are symmetrical and provide a uniform structure for measurement.

##### Experiment 2: DO mass transfer as a function of air flow rate

Aeration of a liquid by a photobioreactor can be characterized as the interfacial mass transfer between the liquid and the gas interface. Therefore, we assessed DO mass transfer in the reactor chamber using gas flow rates ranging from 2.5 to 15 L/min with 2.5 L/min increments. From these trials, we were able to calculate saturation time and mass transfer rates (for details on calculation see below). The true mass transfer coefficient is difficult to measure and estimate independently; however, the effective interfacial area can be mathematically determined using different heuristics techniques [32]. We therefore explored the most common models typically applied to this problem in order to find the best fit (Table 2).

**Table 2:** Summary of common overall mass transfer coefficient equation for bubble column

Author	Equation
Lewis (1924) [32]	${k}_{L}=\frac{{D}_{AB}}{{\updelta }_{\mathrm{L}}}$
Higbie (1935) [25]	$\stackrel{-}{{k}_{L}}=2\sqrt{\frac{{D}_{V}}{\pi {t}_{T}}}=1.13\sqrt{\frac{{D}_{V}}{{t}_{T}}}$
Danckwarts (1955) [17]	${k}_{L}={{(D}_{AB}s)}^{0.5}$
Linek (2005) [33]	${k}_{L}=0.448{\left(\frac{P\rho }{V}\right)}^{0.25}{\left(\frac{{D}_{L}}{r}\right)}^{0.5}$
Kawase and Moo Young (1991) [29]	${k}_{L}a=\frac{7CF\sqrt{{D}_{A}}{u}_{g}{\rho }_{L}^{0.85}\sqrt{g}}{{\mu }_{B}^{0.25}{\sigma }^{0.6}{d}_{c}^{0.17}}$
Ferreira (2012) [20]	${k}_{L}a=3372\sqrt{\frac{{D}_{L}}{\pi }\sqrt{\frac{{u}_{G}^{1.87}g}{{\mu }_{l}{\mu }^{0.24}}}}$
Calderbank (1958) [8]	${k}_{L}=0.422{N}_{\mathrm{SC}}^{-\frac{2}{3}}{\left(\frac{\Delta \rho {\mu }_{c}g}{{\rho }_{c}^{2}}\right)}^{1/3}$
Levich (1962) [31]	${k}_{L}=\frac{{D}^{0.5}{\rho }^{0.5}}{{\sigma }^{0.5}{v}_{o}^{1.5}}$
Godbole (1984) [21]	${k}_{L}=0.146{({j}_{g} }^{0.71}{)(v}^{-0.49})$
Heijnen (1984) [24]	${k}_{L}a=0.32{j}_{g}^{0.7}$
Kawase (1987) [28]	$\mathrm{Sh}=0.63*\frac{1}{\sqrt{\pi }}\sqrt{1.07}{\mathrm{Sc}}^\frac{1}{2}{\mathrm{Re}}^\frac{3}{4}{\mathrm{Fr}}^\frac{7}{60}{\mathrm{Bo}}^\frac{3}{5}$
Nakanoh (1980) [37]	${k}_{L}=\frac{0.09{D}_{C}}{{d}_{\mathrm{pipe}}}{\mathrm{Sc}}^\frac{1}{2}{\mathrm{Bo}}^\frac{3}{4}{\mathrm{Ga}}^{0.39}\mathrm{Fr}$

##### Experiment 3: bubble pattern characterization and void fraction

To analyze bubble column characteristics and measure void fraction as a function of gas flow rate, a Photron high-speed digital video camera was used to capture the regime of the two-phase flow. Using custom MATLAB code, the void fraction at a given flow rate was determined based on individual frame analysis. To increase the system’s ability to accurately read the void fraction, the image was captured at multiple locations within the reactor. These patterns were chosen because they are symmetrical and provide a uniform structure for measurement. Since the camera captured the image and showed it in two dimensions, the area of the gas portion over the total area would be equivalent to the void fraction of the system. The MATLAB program first converted the captured image to a binary image. The white color represents gas and the black color represents water. By dividing the white colored pixels of the image over the total pixels of the image, the void fraction of the system can be found. The void fraction is strongly related to the bubble size and the flow pattern.

#### Membrane cartridge mass transfer experiments

The photobioreactor we developed is designed to effectively transfer nutrients from the wastewater stream into the algal bioreactor column (Fig. 1). The membrane cartridge provides the interface between these two compartments and in Experiment 4 we analyzed mass transfer rates in real time, using artificial salt water (Instant Ocean™ SS15-10) gradients. In Experiment 5, we focused on the impact of the membrane cartridge geometry on mass transfer rate. The artificial sea salt Instant Ocean™ is a complex mixture of ions and, therefore, the relationship between concentration and conductivity had to be determined empirically using a conductivity probe (Atlas Scientific K 1.0 E.C. probe) Each probe was calibrated using eight solutions of known concentration of Instant Ocean using serial dilutions which were used to plot a calibration curve; this curve was then added to the LabVIEW data acquisition system.

##### Experiment 4: optimization of air, water flow and concentration gradient for maximizing mass transfer rates

We developed a comprehensive experimental matrix to determine the optimal operating conditions that produce the highest mass transfer rate across a range of water and air flow rates as well as salt gradients (Table 3). For each test, the concentration of the Instant Ocean salts was adjusted. Additionally, the number of membranes in the reactor chamber was 15 (50% capacity). The membranes used were Fisher Scientific Cellulose Dialysis tubing with a quoted pore diameter of 4.8 nm (S25645B). The reactor was operated for 1 h for each test.

**Table 3:** Operating conditions tested for salt water trials

Total water flow rate (LPM)	3, 6, 9, 12, 15
Air injection flow rate (LPM)	0.5, 5, 14
Reservoir initial instant ocean concentration (ppt)	1, 2, 5

##### Experiment 5: impact of membrane cartridge geometry on mass transfer rate

We manipulated the number of membranes in a separate set of trials in order to gain insight into the effect of the membrane volume ratio on the mass transfer rate. For these experiments the reactor was operated at 9 LPM of saltwater flow with 5 LPM of air injection and with a saltwater concentration gradient of 1 ppt. The configurations for each test are shown in Fig. 1. A volume ratio was used to quantify the volume of membrane cartridges used in the reactor per volume of algae chamber fluid defined as:

2 $$VR= \frac{{V}_{\mathrm{m}}}{{V}_{\mathrm{RB}}},$$

where, ${V}_{\mathrm{m}}$ is the filled volume of membranes being used and ${V}_{\mathrm{RB}}$ is the volume of water in the reactor body.

##### Experiment 6: *Dunaliella tertiolecta* growth experiment

We tested our prototype by measuring growth of *D. tertiolecta* in the reactor compartment in response to nutrients that we added to the reservoir. *D. tertiolecta* (UTEX 999) was obtained from Culture Collection of Algae at The University of Texas at Austin (UTEX). Cultures were grown over time in a growth chamber within the University of Guelph Phytotron at a temperature of 20 °C and a light intensity of 70 uM/m^2^/s. Cultures were growth in 250-mL Erlenmeyer flasks and were diluted weekly to get a transparent light green color which equals to approximate concentration of ~ 5000 cells/100 µL. During this experiment, algal cells were added directly to the 25 L algal compartment at an initial concentration of ~ 10 cells/100 uL with a concentration of *F*/2 equal to that of Guillard’s recipe [23]. Cells were then mixed using 4 LPM of air for an hour, after this 3 × 10 mL samples were taken in 15 mL falcon tubes (Fisher Scientific #14-959-53A). The algae was grown in the tank with an air flow rate of 4 LPM and 65 uM/m^2^/s. 36% formaldehyde was added to the 10 mL bioreactor samples to get a final concentration of 4% in order to immobilize cells. Samples were left to incubate in the dark at room temperature; these samples were labeled as “day 0”. The next day (day 1), samples were collected using the same procedure. However, 1 h of incubation in the dark with 4% formaldehyde, 100 uL of all sample (i.e. day 0 and 1) were plated onto a 96-well plate (Fisher Scientific # 269787) with each of the three daily sample representing two wells on the multi-well plate. The plate was then centrifuged at 2300 rpm for 10 min and then imaged on a Nikon Ti2 at 2× DIC and CY5. Automated counts using the Nikon Ti2 were conducted. The above steps were repeated for day 2 and 3 with images for day 2/3 cell counts being taken on day 3.

We also measured nutrient transfer through the membrane by loading ammonium into the reservoir and measuring ammonium concentration after 5 h in the algal compartment. Target ammonium concentrations were 50 mg/L and equilibrium between the reservoir and algae compartment would, therefore, be 25 mg/L. Water samples were taken prior to ammonium addition (blank) as well as from the reservoir and algae compartment prior to the experiment (initial) and after 5 h (final). Ammonium was measured using HACH tests (#2395366 and #2395566) following manufacturers instructions. A total of 4 replicates were completed for each datapoint.

### Signal processing and data analysis

#### Oxygen mass transfer calculations (Experiments 1–3)

The mass transfer coefficient ${k}_{L}a$ was based on the configuration and the hydrodynamics of the bioreactor system. The value of the mass transfer coefficient was presumed to be the instant mass balance of the solute and is represented as follows:

3 $$\frac{\mathrm{d}{C}_{L}}{\mathrm{d}t}={k}_{L}a*\left({C}_{\mathrm{s}}-{C}_{L}\right),$$

where ${C}_{L}$ is the concentration of the dissolved oxygen in the water at time $t$, and ${C}_{\mathrm{S}}$ is the concentration of the dissolved oxygen at a saturated state. The bioreactor setup is an adiabatic system and we therefore assumed that the ambient temperature was constant at (21 °C). The saturated dissolved oxygen was always set to be 9.1 mg/L. As well, ${k}_{L}a$ is the overall mass transfer coefficient of the bioreactor system.

Adopting the work of Boyd [6] and Colt [13], the mass transfer coefficient was calculated through linear regression analysis. With the integration of Eq. (3), the overall mass transfer coefficient, ${k}_{L}a,$ relative to the overall volume in the bioreactor is expressed as follows:

4 $${k}_{L}a=\mathrm{ln}\left(\frac{{C}_{\mathrm{s}}-{C}_{0}}{{C}_{\mathrm{s}}-{C}_{L}}\right)*\frac{1}{t}.$$

${C}_{0}$ is the initial concentration of the dissolved oxygen in the water. The mass transfer coefficient can be further approximated by multiplying it with a correction factor due to the water temperature. The resulting relationship is expressed as follows:

5 $$k_{L} a = \left( {k_{L} a} \right)_{20} \theta^{T - 20} ,$$

where $\theta$ is the correction factor of the temperature and is always set as 1.024, as all experiments were conducted at standard temperature. T is the temperature of the water during the test.

#### Salt mass transfer calculation (Experiments 4 and 5)

To evaluate the mass transfer through the membrane stack, the slope of the conductivity measured in the algae reactor was used. As air bubble injection introduced noise into the conductivity measurements, signals were processed in MATLAB using an upper and lower envelope of the sampled conductivity and we extracted the upper envelope for further processing. This slope (mass transfer rate, *J* (Eq. 9)—*J* is the measured salt transfer rate, $\Delta x$ is the membrane thickness and *A* is the surface area of exposed membrane in the cartridge), was then used to calculate the diffusivity constant, *D* (Eq. 9), which is a function of the concentration gradient, membrane surface area, and water velocity at the membrane surface (Eqs. 6–9). *D* was then used to determine the Sherwood number (Eq. 10—$d$ is the tube diameter and $v$ is the average fluid velocity) and the Reynolds number (Eq. 11—$\rho$ is the water density and $\mu$ is the fluid viscosity), two non-dimensional parameters, allowing unlimited scaling of the reactor prototype:

6 $$\Delta {C}_{\mathrm{in}}= {C}_{\mathrm{initial},\mathrm{ Res}}-{C}_{\mathrm{initial},\mathrm{Col}},$$

7 $$\Delta {C}_{\mathrm{out}}= {C}_{\mathrm{final},\mathrm{ Res}}-{C}_{\mathrm{final},\mathrm{Col}},$$

8 $${\Delta C}_{\mathrm{mean}}= \frac{{\Delta C}_{\mathrm{in}}-{\Delta C}_{\mathrm{out}}}{\mathrm{ln}\left(\frac{{\Delta C}_{\mathrm{in}}}{{\Delta C}_{\mathrm{out}}}\right)},$$

9 $$D= \frac{J\Delta x}{A{\Delta C}_{\mathrm{mean}}},$$

10 $$\mathrm{Sh}= 1.62{\left(\frac{dv}{D}\right)}^\frac{1}{3},$$

11 $$\mathrm{Re}= \frac{\rho vd}{\mu }.$$

#### Growth characteristics for *D. tertiolecta* (Experiment 6)

We assessed growth characteristics and biomass production in our reactor using the green alga *D. tertiolecta*. We used previously published data [45], to estimate dry biomass production based on cell growth and duplication events from our experiment. We also calculated ammonium mass transfer as representative for nutrient transfer across the membrane due to the ability to easily and quickly quantify ammonium in our experiments.

## Data Availability

Not applicable.
